# The effectiveness of Corticosteroids on mortality in patients with acute respiratory distress syndrome or acute lung injury: a secondary analysis

**DOI:** 10.1038/srep17654

**Published:** 2015-12-02

**Authors:** Zhongheng Zhang, Lin Chen, Hongying Ni

**Affiliations:** 1 Department of critical care medicine, Jinhua municipal central hospital, Jinhua hospital of Zhejiang university, Zhejiang, P.R. China

## Abstract

The development of acute respiratory distress syndrome (ARDS) is associated with dys-regulated inflammation. Since corticosteroids are potent anti-inflammatory drugs, they are thought to be beneficial for ARDS patients. The study aimed to investigate the effectiveness of corticosteroids on mortality outcome in ARDS patients. The study was a secondary analysis of a prospective randomized controlled trial (NCT00979121). ARDS patients with invasive mechanical ventilation were enrolled. Corticosteroids use was defined as IV or PO administration of corticosteroids totaling more than 20 mg methylprednisolone equivalents during one calendar day. Missing data were handled using multiple imputation technique. Multivariable model was built to adjust for confounding covariates. A total of 745 patients were enrolled, including 540 survivors and 205 non-survivors. Patients in the non-survivor group were more likely to use corticosteroids (38% vs. 29.8%; p = 0.032). After adjustment for other potential confounders, corticosteroids showed no statistically significant effect on mortality outcome (OR: 1.18; 95% CI: 0.81–1.71). Furthermore, we investigated the interaction between corticosteroid use and variables of vasopressor and PaO2. The result showed that there was no significant interaction. In conclusion, the study failed to identify any beneficial effects of corticosteroids on mortality outcome in patients with ARDS.

Acute respiratory distress syndrome (ARDS) is commonly seen in the intensive care unit (ICU), with an estimated incidence around 20% to 50% depending on different study populations^1^,^2^. Development of ARDS or its less severe form acute lung injury (ALI) has been associated with adverse outcome. Therefore, strenuous effort has been done to investigate the treatment of ARDS. Although varieties of interventions such as protective ventilation, negative fluid balance, activated protein C and statin has been thought to be clinically useful for outcome improvement, none of them was supported by strong evidence.

Pathophysiologically, the development of ARDS is associated with dys-regulated inflammation, interstitial and alveolar edema, infiltration of cells into alveolar space and endothelial injury^3^,^4^,^5^. Corticosteroids are potent anti-inflammatory drugs that act primarily by down-regulating proinflammatory cytokines such as interleukins 1a, 1b, 2 and 3. Thus corticosteroids are thought to be effective in improving clinical outcomes of ARDS patients^6^.

Several studies have been conducted to investigate the effectiveness of corticosteroids in ALI and/or ARDS. These results are conflicting^7^,^8^,^9^, and the sample sizes are usually small. For example, Meduri GU and coworkers^8^ reported that methylprednisolone was able to ameliorate systemic inflammation response, resulting in significant improvement in pulmonary and extrapulmonary organ dysfunction and reduction in duration of mechanical ventilation and ICU length of stay. However, the study enrolled less than 100 subjects, which was subject to sampling error. In some studies, the effectiveness of corticosteroids in ARDS was only addressed in subgroup analysis. Several meta-analyses reviewed these studies and concluded that there were significant heterogeneity in component trials and the benefits of corticosteroid needs further investigations^10^,^11^,^12^. The present study aimed to investigate the effectiveness of low-dose corticosteroids on mortality outcome in ARDS patients.

## Methods

The study was a secondary analysis of a prospective randomized controlled trial (NCT00979121). The dataset was collected from 44 enrolling hospitals in the national heart, lung and blood institute ARDS clinical trial network. The original study was approved by the institutional review board at each participating center^13^. The secondary data analysis was approved by the institutional review board of Jinhua municipal central hospital. The study was carried out in accordance with the Declaration of Helsinki. Written informed consent was obtained from all subjects in the original study. Patient records/information was anonymized and de-identified prior to analysis.

### Study population

Patients were eligible if they fulfilled following criteria: (1) invasive mechanical ventilation; (2) a partial pressure of arterial oxygenation to fraction of inspired oxygen ratio of less than 300 mmHg; (3) bilateral infiltrates on chest radiography; (4) without evidence of left atrial hypertension. All these criteria must be fulfilled within 24 hours after randomization. Exclusion criteria included: (1) presence of ARDS for more than 48 hours; (2) chronic conditions that impair weaning from mechanical ventilation, or compromise adherence to study protocol; (3) inability to obtain consent^13^.

### Corticosteroid use

Corticosteroids use was defined as IV or PO administration of corticosteroids totaling more than 20 mg methylprednisolone equivalents during one calendar day. 20 mg methylprednisolone equals to 3.75 mg dexamethasone, 25 mg prednisone and 100 mg hydrocortisone. Corticosteroids were recorded during day 1 to day 7. For patients who died or discharged before day 7, this was recorded as missing values.

### Study endpoint

In the original study, patients were followed up until death or day 90 after enrollment. The study endpoint was categorized into three conditions: (1) Home with unassisted breathing (UAB): the patient is discharged home with unassisted breathing. The home here is defined as the place the patient lived prior to this episode of hospital admission; (2) death: the patient died prior to home discharge or died prior to achieving unassisted breathing at home for 48 hours; (3) Other: neither of the above condition was met. For example, if a patient went home on assisted breathing and has not achieved unassisted breathing for 48 hours, continues on assisted breathing, or has been transferred to another facility, other than home, on unassisted breathing. Conditions (1) and (3) were combined as survivors and condition (2) was regarded as non-survivors.

### Data extraction

The original study examined the effectiveness of rosuvastatin on mortality outcome. However, the rosuvastatin showed neutral effect and we did not consider the effect of rosuvastatin on mortality. Demographics such as gender, age and ethnics were reported. The type of ICU including medical intensive care unit (MICU), surgical intensive care unit (SICU), cardiac SICU, coronary care unit (CCU), Neuro ICU, burn care unit, trauma ICU and mixed MICU/SICU were obtained. Other included variables were the number of quadrants with infiltrates on chest X-ray; suspected or documented infection site; vasopressor use, urine output, partial pressure of arterial oxygen (PaO2), central venous pressure (CVP), creatinine kinase (CK), alanine aminotransferase (ALT), C-reactive protein (CRP) and APACHE III score. All these variables were recorded within 24 hours after enrollment.

### Statistical analysis

#### Univariate analysis

Variables were expressed as mean (SD) or the frequency as appropriate. Comparisons between survivors and non-survivors were performed by using student t test for continuous variables, or Chi-square test for categorical variables.

#### Multiple imputation

Because missing values were common in the dataset, we employed multiple imputation (IM) to address the problem of information loss due to listwise deletion of observations in estimation^14^,^15^. The main appealing features of MI included (1) the ability to perform varieties of completed-data analyses using existing statistical methods; and (2) separation of the imputation step from the analysis step. To reduce the sampling error due to imputations, we set the number of imputations to be 20 as recommended by some authors^16^.

Variables to be incorporated in the logistic regression model for completed-data analysis were gender, type of ICU, ethnic, source of infection, APACHE III, vasopressor use on day 0, CVP, the number of quadrats of infiltrates, CRP, CK, ALT, urine output. These variables were empirically proven or thought to be associated with mortality outcome^17^,^18^,^19^,^20^,^21^,^22^. Variables included in APACHE III as components were not used in multivariable model to avoid the potential problem of multicollinearity. We examined these variables with STATA command *codebook*, which showed that APACHE III, CVP, the number of quadrats of infiltrates, CRP, CK and urine output contained missing values.

We followed several steps to perform the MI procedure: (1) the dataset was declared as marginal long style, because it was a memory-efficient style. (2) All variables with missing values were registered as imputed variable. (3) multivariate normal regression model was used for the imputation procedure. Variables employed for imputation were those obtained within 24 hours after initiation of the study including mortality outcome, age, gender, source of admission, type of patients, chronic dialysis, vasopressor use, temperature, blood pressure, heart rate, respiratory rate and infection site. There were no missing values for these variables. We created 20 imputations to reduce the simulation (Monte Carlo) error. The seed was arbitrarily set to be 29390 for reproducibility. (4) We fitted the logistic regression using the *mi estimate* prefix command.

#### Model building strategy

Because the purpose of the study was to adjust for the effectiveness of corticosteroid, we included as much covariate as possible. Variables to be incorporated in the logistic regression model for completed-data analysis were gender, type of ICU, ethnic, source of infection, APACHE III, vasopressor use on day 0, CVP, the number of quadrats of infiltrates, CRP, CK, ALT, urine output and PaO2. Because patients on shock requiring vasopressors and/or severe hypoxia may benefit from the use of corticosteroids, we explored interactions between them. Because the aim of the study was to investigate the effectiveness of corticosteroids on ARDS patients (e.g. the predictive value of the model was not so important), we included all covariates that were thought to be associated with mortality outcome. Model discrimination and calibration were assessed by graphical presentation of observed and predicted outcomes, as well as the receiver operating characteristic curve (ROC). Also we reported the Homser-Lemeshow goodness-of-fit statistic for assessment of model fit^23^.

All statistical analyses were performed by using STATA 13.1 (College Station, TX 77845, USA). Statistical significance was considered at p < 0.05.

## Results

**Figure 1:**
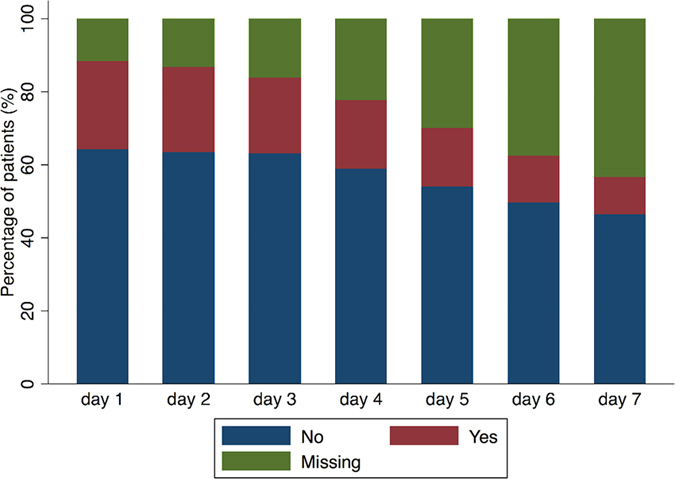
Graphical presentation of patients receiving corticosteroids, those without receiving corticosteroids and those with missing data. The proportion of patients with missing data increased from day 1 to day 7, which was attributable to ICU discharge or death (the end of follow up).

A total of 745 patients were enrolled, including 540 survivors and 205 non-survivors. Patients in the non-survivor group were more likely to use corticosteroids (38% vs. 29.8%; p = 0.032). As expected, more patients in the non-survivors required vasopressor than survivors (63.4% vs. 51.5%; p = 0.003). Other variables such as gender, ethnic, ICU location, the number of quadrants with infiltrates and infection site were not significantly different between survivors and non-survivors (Table 1). Survivors were significantly younger (52.00 ± 15.92 vs. 59.71 ± 16.17 years, p < 0.001) and had lower values of APACHE III (88.42 ± 26.86 vs. 106.72 ± 27.30; p < 0.001) than non-survivors. CK value was higher in survivors than in non-survivors (244.88 ± 430.80 vs. 151.34 ± 327.31 U/l; p = 0.01). Survivors had significantly greater volume of 24-hour urine output than non-survivors (1668.75 ± 1235.44 vs. 1437.33 ± 1226.21 ml; p = 0.02). Other continuous variables such as CVP, ALT, CRP and PaO2 were not statistically different (Table 2). Missing values in corticosteroid use increased with time (Fig. 1). There were 10% missing values on day 1 and this figure monotonously increased to 40% on day 7.

**Figure 2:**
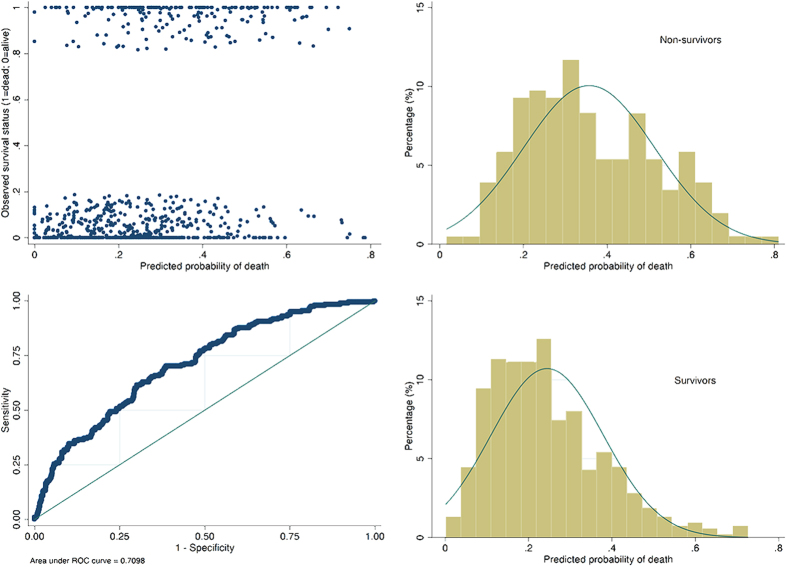
Four diagnostic plots to describe discrimination in a model fit with an area under operating characteristics curve of 0.71. The plot of jittered outcome versus estimated probability of death showed that survivors were morel likely to appear below 0.4. However, non-survivors were normally distributed with the mean value at somewhere between 0.3 and 0.4. The Hosmer-Lemeshow chi2 statistic was 4.89 (p  =  0.7689).

After careful examination of all variables, we found that APACHE III, CVP, the number of quadrats with infiltrates, CK, CRP, PaO2 and urine output had missing values (Table 3). MI was performed to impute missing values and all missing values were imputed. After adjustment for other potential confounders, corticosteroids showed no statistically significant effect on mortality outcome (OR: 1.18; 95% CI: 0.81–1.71). As expected, APACHE III was a significant predictor of mortality (OR: 1.02; 95% CI: 1.02–1.03). In multivariable model, CK continued to be an important protector of mortality outcome (OR: 0.999; 95% CI: 0.998–0.9998), but the effect size was marginal and of limited clinical relevance (Table 4). Furthermore, we investigated the interaction between corticosteroid use and variables of vasopressor and PaO2. The result showed that there was no significant interaction (Table 5). Discrimination power of the model was moderate in predicting mortality outcome (area under curve was 0.71, Fig. 2).

### Propensity score matching

**Figure 3:**
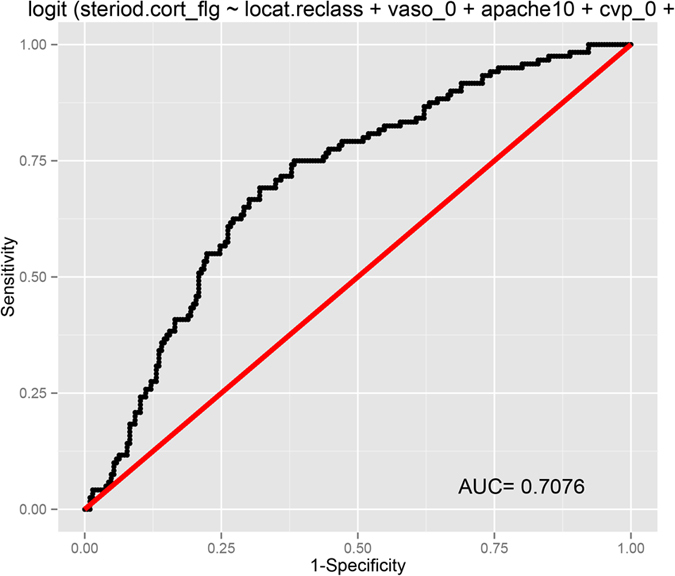
Receiver operating characteristics curve showing the discrimination power of the logistic regression model in predicting corticosteroid use. The area under curve was 0.71.

Table 6 shows the clinical characteristics of patients with and without corticosteroid treatment. The results showed that patients treated in MICU were more likely to receive corticosteroids (73.6% vs. 57.7%, p < 0.001). Patients on vasopressor on the first day were more likely to receive corticosteroids (62.3% vs. 51.2%, p = 0.005). Patients receiving corticosteroids were more critically ill with higher APACHE III scores (p < 0.001). There was no difference between patients with and without corticosteroids in gender, ethnics, infection site, CVP, CK, PaO_2_ and age. In multivariate model, the SICU appeared to be a factor against use of corticosteroids (OR: 0.22, 95% CI: 0.08–0.53). Use of vasopressor was associated with higher probability of corticosteroids use (OR: 1.91, 95% CI: 1.10–3.36). Urine output and CVP were not independently associated with corticosteroids use (Table 7). Overall the model had moderate discrimination in predicting corticosteroid use (AUC = 0.71, Fig. 3).

**Figure 4:**
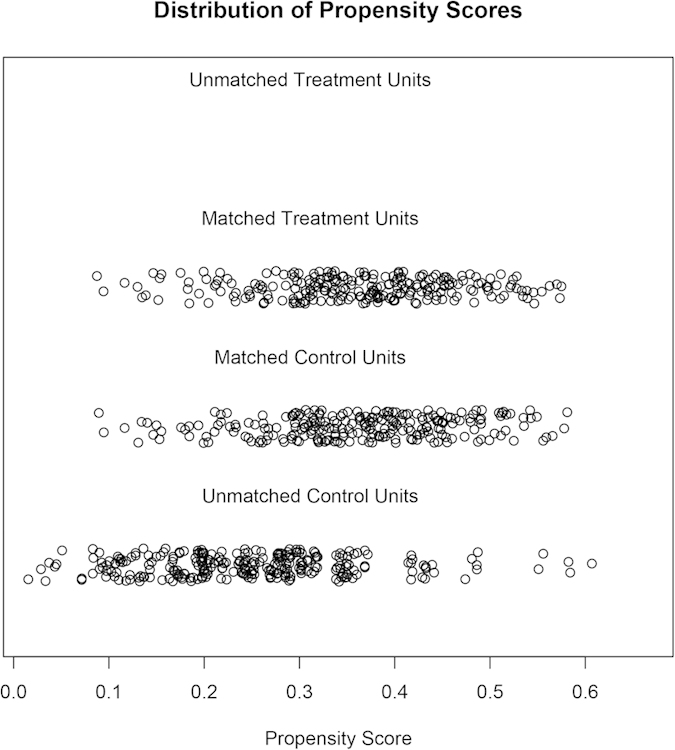
Distribution of propensity scores. All treated patients were matched to the untreated patients.

CVP and urine output were excluded from logistic regression model for generate propensity score. Number of quadrants with infiltrates was also excluded because this variable has too many missing values and it was only marginally significant. We used nearest matching strategy. The propensity scores of individual patients in all patients before and after matching were shown in Fig. 4. A total of 239 treated patients were matched to 239 control patients. The remaining 267 patients in the control group were not matched. In the matched cohort, the mortality risk in the corticosteroid group was not significantly different from that in the control group (48.1% vs. 54.5%, p = 0.231).

## Discussion

The study failed to identify any beneficial effects on mortality outcome in patients with ARDS. The study was a secondary analysis of a prospectively collected dataset. In this cohort, corticosteroids were more likely to be given to non-survivors. The most plausible causal relationship is that more critically ill patients were more likely to use corticosteroids. Although there was no strong evidence supporting the use of corticosteroids in ARDS patients, physicians are still prescribing corticosteroids for them as an alternative to conventional therapies in the hope that corticosteroids may ameliorate pulmonary edema. The American College of Critical Care Medicine issued a recommendation that glucocorticoids should be considered in the management strategy of patients with early severe ARDS^24^. In this background, the present study confirmed the futility of corticosteroids use in ARDS patients.

The use of corticosteroids in ARDS patients was not novel and several small studies have been conducted to address this issue. The first study conducted in early 1980s by Bernard and coworkers^25^. They investigated the high-dose corticosteroids on mortality outcome in ARDS patients. The study stopped early after enrollment of 99 patients because of the futility of the study drug. Because of the negative result of the study, the interests on this topic waned by the end of 1980s. However, the study by Annane and colleagues renewed the interests on corticosteroids, in which they found that corticosteroids were able to reduce the risk of death in patients with illness-related adrenal insufficiency (53% vs 63%; P = 0.04). Although the study population was sepsis, there was substantial number of patients with ARDS, accounting for 59% of the whole population^7^,^26^. However, the result could not be replicated in other studies^9^,^10^,^12^,^27^. Overall, the main findings in the literature were consistent with our result.

One limitation of the study was that there were some missing values in the dataset. We used MI to address the problem of information loss due to missing values. Missing data is common in publically available dataset and reflect the quality of the establishment of a dataset. In our dataset, the proportion of missing values can be as much as one third of a variable. If multivariable regression model was built by conventional method (listwise deletion), the number of observations remained in the model will be extremely small. There are other techniques for handling missing data, such as complete case analysis, overall mean imputation, and the missing-indicator method. However, these techniques are found to be less reliable than MI^15^,^28^. The other limitation of our study was that other clinically interesting outcomes were not investigated. These included ICU length of stay, organ failure free days and the duration of mechanical ventilation. Although these secondary study end points may not necessarily translate to mortality benefit, they are important from the perspective of cost-effectiveness. For example, if the duration of mechanical ventilation or ICU length of stay can be shortened, the medical cost can be substantially reduced. There are a few evidences supporting the beneficial effect of corticosteroids in improving these secondary outcomes. Meduri GU and coworkers reported that Methylprednisolone-induced down-regulation of systemic inflammation was associated with significant improvement in extrapulmonary and pulmonary organ failure, as well as the reduction in duration of mechanical ventilation and ICU length of stay^8^. The result was confirmed by subsequent systematic review^10^.

In conclusion, the study failed to identify any beneficial effect of corticosteroids on mortality outcome. Although non-survivors were more likely to use corticosteroids, the effect disappeared after adjustment by the severity of illness. The use of multiple imputation technique helped to improve the estimation of the effect size by preserving all useful information.

## Additional Information

**How to cite this article**: ZHANG, Z. *et al.* The effectiveness of Corticosteroids on mortality in patients with acute respiratory distress syndrome or acute lung injury: a secondary analysis. *Sci. Rep.* **5**, 17654; doi: 10.1038/srep17654 (2015).

## Figures and Tables

**Table 1:** Comparison between survivors and non-survivors for categorical variables.

	Survivors		Non-survivors		Total	
	No.	percentage	No.	percentage	No.	percentage
Corticosteroids use^**§**^						
No	379	70.2%	127	62.0%	506	67.9%
Yes	161	29.8%	78	38.0%	239	32.1%
Total	540	100.0%	205	100.0%	745	100.0%
Pearson chi2(1) = 4.6235 Pr = 0.032						
Gender						
Male	260	48.1%	105	51.2%	365	49.0%
Female	280	51.9%	100	48.8%	380	51.0%
Total	540	100.0%	205	100.0%	745	100.0%
Pearson chi2(1) = 0.5609 Pr = 0.454						
Ethnic						
Hispanic or Latino	66	12.2%	20	9.8%	86	11.5%
Others	474	87.8%	185	90.2%	659	88.5%
Total	540	100.0%	205	100.0%	745	100.0%
Pearson chi2(1) = 0.8850 Pr = 0.347						
Location						
MICU	337	62.4%	131	63.9%	468	62.8%
SICU	27	5.0%	6	2.9%	33	4.4%
Cardiac SICU	3	0.6%	2	1.0%	5	0.7%
CCU	5	0.9%	2	1.0%	7	0.9%
Neuro ICU	15	2.8%	2	1.0%	17	2.3%
Burn	6	1.1%	3	1.5%	9	1.2%
Trauma	16	3.0%	3	1.5%	19	2.6%
MICU/SICU	126	23.3%	53	25.9%	179	24.0%
Others	5	0.9%	3	1.5%	8	1.1%
Total	540	100.0%	205	100.0%	745	100.0%
Pearson chi2(8) = 6.2595 Pr = 0.618						
Infection site						
Thorax	386	71.5%	147	71.7%	533	71.5%
Abdomen	47	8.7%	18	8.8%	65	8.7%
Skin or soft tissue	24	4.4%	5	2.4%	29	3.9%
Bacterial meningitis	2	0.4%	2	1.0%	4	0.5%
Urinary tract	38	7.0%	13	6.3%	51	6.8%
Central line	1	0.2%	1	0.5%	2	0.3%
Osteomyelitis	2	0.4%	2	1.0%	4	0.5%
Confirmed Swine Influenza A	1	0.2%	0	0.0%	1	0.1%
Others	38	7.0%	16	7.8%	54	7.2%
Suspected infection	1	0.2%	1	0.5%	2	0.3%
Total	540	100.0%	205	100.0%	745	100.0%
Pearson chi2(9) = 5.1841 Pr = 0.818						
Vasopressor use						
No	262	48.5%	75	36.6%	337	45.2%
Yes	278	51.5%	130	63.4%	408	54.8%
Total	540	100.0%	205	100.0%	745	100.0%
Pearson chi2(1) = 8.5413 Pr = 0.003						

^§^Patients were considered to have corticosteroid use when they received IV or PO corticosteroids totaling >20 mg methylprednisolone equivalents on one calendar day during first 7 days.

**Table 2:** Comparison between survivors and non-survivors for continuous variables.

	Survivors		Non-Survivors		P value
	Median	IQR	Median	IQR	
Age	53	41–63	60	50–70	<0.001
APACHE III	86.5	69–106	103	89–124	<0.001
CVP	11	8.5–11.0	11	8–15	0.63
CK	103	39–265	66	31–138	0.01
ALT	27	17–44	25	17–38	0.91
CRP	22.5	12.6–30.8	21.4	12.9–31.6	0.90
Urine output (24 hours)	1388	799–2217	1062	579–2050	0.02
Number of quadrants with infiltrates	4	3–4	4	3–4	0.018
PaO2	84	70–106	82	69–104	0.62

Abbreviations: SD: standard deviation; APACHE: acute physiology and chronic health evaluation; CVP: central venous pressure; CK: creatine kinase; CRP: C-reactive protein; ALT: alanine aminotransferase; PaO2: partial pressure of arterial oxygen; IQR: interquartile range.

**Table 3:** The result of multivariate imputation by using multivariate normal regression model.

Variables	Observations per m			
	Complete	Incomplete	Imputed	Total
APACHE III	707	38	38	745
CVP	456	289	289	745
Number of quadrats with infiltrates	560	185	185	745
CK	743	2	2	745
CRP	699	46	46	745
PaO2	733	12	12	745
Urine output	741	4	4	745

Abbreviations: APACHE: acute physiology and chronic health evaluation; CVP: central venous pressure; CK: creatine kinase; CRP: C-reactive protein; PaO2: partial pressure of arterial oxygen.

Note: right-hand-side variables (variables used for multiple imputation) have missing values; model parameters estimated using listwise deletion. Complete + incomplete = total; imputed is the minimum across m of the number of filled-in observations.

**Table 4:** Adjustment of confounding factors with multivariate regression model.

Mortality outcome	Odds Ratio	Lower limit of 95% CI	Upper limit of 95% CI	P > t
Corticosteroids	1.18	0.81	1.71	0.396
Female (male as reference)	0.86	0.60	1.22	0.393
Location				
SICU	0.81	0.30	2.14	0.666
Cardiac SICU	1.13	0.15	8.72	0.908
CCU	0.89	0.15	5.16	0.898
Neuro ICU	0.75	0.16	3.65	0.726
Burn	2.05	0.36	11.55	0.418
Trauma	0.64	0.16	2.60	0.531
MICU/SICU	1.20	0.79	1.81	0.402
Others	1.42	0.30	6.70	0.656
Other ethnic (Hispanic or Latino as reference)	1.28	0.72	2.26	0.406
Infection site				
Abdomen	0.88	0.47	1.64	0.691
Skin or soft tissue	0.53	0.17	1.66	0.276
Bacterial meningitis	2.26	0.26	19.78	0.461
Urinary tract	0.83	0.41	1.69	0.613
Central line	1.79	0.11	29.82	0.685
Osteomyelitis	1.61	0.20	12.63	0.652
Confirmed Swine Influenza A	1			
Others	0.89	0.45	1.77	0.744
Suspected infection (no site specified)	4.19	0.22	78.35	0.338
APACHE III	1.02	1.02	1.03	<0.001
Vasopressor use	1.04	0.71	1.54	0.829
CVP	.98	0.94	1.02	0.419
Number of quadrats with infiltrates (with each one increase)	1.22	0.93	1.60	0.142
CK	0.999	0.998	0.9998	0.009
ALT	1.001	0.996	1.006	0.816
CRP	1.00	0.99	1.005	0.713
Urine output	1.00	1.00	1.00	0.843
PaO2	1.00	0.99	1.01	0.839
Constant term	0.03	0.01	0.12	<0.001

Abbreviation: APACHE: acute physiology and chronic health evaluation; CVP: central venous pressure; CK: creatine kinase; CRP: C-reactive protein; PaO2: partial pressure of arterial oxygen.

**Table 5:** Interactions between corticosteroid use and arterial oxygen partial pressure and vasopressor use.

Variables	Odds ratio	Lower limit of 95% CI	Upper limit of 95% CI	p
Interaction between Corticosteroid and Vasopressor				
Corticosteroid	1.32	0.72	2.42	0.361
Vasopressor	1.11	0.70	1.76	0.654
Corticosteroid × Vasopressor	0.83	0.39	1.76	0.623
Interaction between Corticosteroid and partial pressure of arterial oxygen				
Corticosteroid	1.26	0.40	3.98	0.696
PaO2	1.00	0.99	1.01	0.924
Corticosteroid × PaO2	1.00	0.99	1.01	0.902

Note: interaction terms were assessed in independent models by adjusting for the same covariates. Corticosteroid and vasopressor were indicator variables, and PaO2 was continuous variable.

Abbreviations: PaO2: partial pressure of arterial oxygen; CI: confidence interval.

**Table 6:** Characteristics of patients with and without corticosteroids.

Variables	No corticosteroids (n = 506)	Corticosteroids use (n = 239)	P
Gender (male, %)	251 (49.6)	114 (47.7)	0.684
Location (N, %)			<0.001
Burn	8 (1.6)	1 (0.4)	
Cardiac SICU	4 (0.8)	1 (0.4)	
CCU	4 (0.8)	3 (1.3)	
MICU	292 (57.7)	176 (73.6)	
MICU/SICU	137 (27.1)	42 (17.6)	
Neuro ICU	13 (2.6)	4 (1.7)	
Others	3 (0.6)	5 (2.1)	
SICU	29 (5.7)	4 (1.7)	
Trauma	26 (3.2)	3 (1.3)	
Ethnic (N, %)			1
Hispanic or Latino	58 (11.5)	28 (11.7)	
Others	448 (88.5)	211 (88.3)	
Infection sites (N, %)			0.713
Abdomen	49 (9.7)	16 (6.7)	
Bacterial meningitis	2 (0.4)	2 (0.8)	
Central line	1 (0.2)	1 (0.4)	
Confirmed Swine Influenza A	1 (0.2)	0	
Osteomyelitis	2 (0.4)	2 (0.8)	
Others	41 (8.1)	13 (5.4)	
Skin or soft tissue	18 (3.6)	11 (4.6)	
Suspected infection	1 (0.2)	1 (0.4)	
Thorax	356 (70.4)	177 (74.1)	
Urinary tract	35 (6.9)	16 (6.7)	
Vasopressor (N, %)	259 (51.2)	149 (62.3)	0.005
APACHE III (median, IQR)	89 (70–109)	96.5 (79–117.2)	<0.001
CVP (mmHg)	11 (8–14)	11 (9–15)	0.176
The number of quadrants with infiltrates (median, range)	4 (0–4)	4 (2–4)	0.037^§^
CK (mmol/l)	91 (40–233)	75 (31–195)	0.336
ALT (mmol/l)	26 (16–41)	27 (17–44)	0.298
CRP (mg/dl)	22.7 (13.5–31.35)	21.2 (10.98–30.60)	0.003
Urine output (24 hours)	1400 (785–2218)	1170 (645.2–1968)	0.063
PaO2 (mmHg)	84 (70–104.5)	83 (68–105)	0.700
Mortality (N,%)	127 (25.1)	78 (32.6)	0.039
Age (years)	55 (42–66)	56 (43–65)	0.866

Abbreviations: IQR: interquartile range; APACHE: acute physiology and chronic health evaluation; CVP: central venous pressure; CK: creatine kinase; CRP: C-reactive protein; PaO2: partial pressure of arterial oxygen.

^§^ statistical test was performed by using Cochran-Armitage trend test.

**Table 7:** Multivariate regression model to predict corticosteroid use.

Variables	Odds ratio	95% confidence interval	p
Locate (MICU as reference)			
Mixed ICU	0.45	0.24–0.83	0.011
SICU	0.22	0.08–0.53	0.002
Vasopressor	1.91	1.10–3.36	0.024
APACHE III (for each 10 points)	1.10	0.998–1.212	0.056
CVP	1.01	0.97–1.07	0.575
Number of quadrants with infiltrates	1.36	0.98–1.92	0.073
CRP	0.98	0.96–0.99	0.036
Urine output (for each 100ml)	1.01	0.99–1.030	0.581

Abbreviations: MICU: medical intensive care unit; ICU: intensive care unit; SICU: surgical intensive care unit; PACHE: acute physiology and chronic health evaluation; CVP: central venous pressure; CRP: C-reactive protein.
